# The role of magnetic resonance imaging in the evaluation of scaphoid fractures

**DOI:** 10.1002/jmrs.316

**Published:** 2019-03-04

**Authors:** Steven B. S. Wong, Wilfred C. G. Peh

**Affiliations:** ^1^ Department of Radiology Sengkang General Hospital Singapore Singapore; ^2^ Department of Diagnostic Radiology Khoo Teck Puat Hospital Singapore Singapore

**Keywords:** magnetic resonance imaging, scaphoid, fracture, radio‐occult

## Abstract

Radiographs are currently accepted as the first‐line modality for the investigation of scaphoid fractures. Early use of magnetic resonance imaging (MRI) in patients with radio‐occult scaphoid fractures has been shown to be beneficial in the management of such patients. Incorporation of early MRI into the management protocol for scaphoid fracture is recommended.

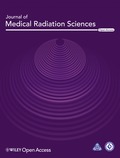

Scaphoid fractures are common, can appear subtle and may be difficult to detect radiographically. A misdiagnosed scaphoid fracture has adverse implications for the patient, in particular the well‐known complication of avascular necrosis of the proximal scaphoid pole due to disruption of its vascular supply. Such affected patients are at risk of suffering pain and disability, as well as developing degenerative joint disease.

Radiographs of the scaphoid have long been the mainstay investigation for diagnosis, due to its low cost and availability. Patients with a positive radiograph for scaphoid fracture are usually promptly treated with immobilisation and if needed, surgery. In patients with a negative radiograph but with high clinical suspicion of a fracture, including pain in the anatomical snuffbox, the time‐honoured management is immobilisation and then repeat radiographs after a 2‐week interval. Prophylactic immobilisation in a cast comes with its own set of challenges for the patients, including difficulty with daily activities, potential loss of function of the hand and time off work in some cases. A negative radiograph at the end of 2 weeks will also mean that the patient will have been inconvenienced for the duration of limb immobilisation.

During the last decade, the increased availability and applications of magnetic resonance imaging (MRI) scanners and decreasing cost of this investigation have opened up new options for the early detection of radiographically occult scaphoid fractures. Kelson et al^1^ compared the early use of MRI in patients with suspected scaphoid fractures to patients with conventional management in a rural setting. While healthcare costs were similar in both groups, the MRI group had lower pain and higher satisfaction scores as well as less time required for immobilisation, treatment and time off work. Given potential societal costs, the authors recommended early MRI*,* from 1 to 3 days of injury, to be the management technique of choice.

A similar approach was recommended by Wijetunga et al^2^ in a study reported in the current issue of Journal of Medical Radiation Sciences. The authors conducted a retrospective review of patients with suspected scaphoid fractures presenting to an urban hospital. Patients who underwent conventional management utilising radiographs taken 2 weeks apart had a longer time to diagnosis of 24.1 ± 17.2 days. Immobilisation was up to 67 days, compared to those who received early CT (computed tomography) or MRI when a significantly reduced diagnosis time of 9.8 ± 5.8 days was achieved. The authors reasoned that continued adherence to the conventional radiographic management protocol may be partly due to the misconception that CT and MRI are costly and less available.

The American College of Radiology (ACR) has recommended MRI as the best second‐line investigation for scaphoid fractures, with radiographs being the first‐line investigation. In the setting of suspected acute scaphoid fracture, when initial radiographs are normal, wrist MRI will be appropriate.^3^ With the clear demonstration of injury‐related bone marrow oedema on the T2‐weighted sequences and the focal hypointense region on T1‐weighted sequences, undisplaced occult scaphoid fractures can be detected early. The presence of other carpal fractures is also more readily identified on MRI.

MRI has a high sensitivity and specificity rate for detection of scaphoid fractures. On MRI, scaphoid fractures are seen as areas with focal bone marrow signal changes or loss of cortical bone continuity. The higher cost of MRI, if performed early, can be justified and offset by the patient receiving early and appropriate treatment, if positive for a fracture. If the MRI was negative for a scaphoid fracture, the patient will avoid unnecessary immobilisation. This was concluded by several papers, including Patel et al^4^ who, in a randomised controlled trial, studied the cost and clinical effectiveness of MRI in patients with occult scaphoid fractures, compared to those who were managed conventionally. They found that using early MRI in occult scaphoid fractures enables marginal cost saving compared to conventional management, may reduce potentially large societal costs of unnecessary immobilisation, and also allows early detection and appropriate treatment of scaphoid and other injuries.

Karl et al^5^ evaluated the cost‐effectiveness of using advanced imaging modalities such as MRI and CT in patients with suspected occult scaphoid fractures. They found that advanced imaging had advantages over cast immobilisation, had lower costs and projected improved health outcomes when compared to cast immobilisation. MRI was also found to be marginally more cost‐effective than CT. The authors concluded that the relatively low cost and high diagnostic accuracy of advanced imaging for suspected scaphoid fractures following negative radiographs represents a cost‐effective strategy for reducing both societal costs and morbidity in this group of patients.

Medical practitioners treating patients with scaphoid fractures should consider modifying their radiograph‐based diagnostic and management protocols to include early MRI. Earlier detection of a radiographically occult fracture will aid appropriate management, be it surgery or immobilisation. Prompt exclusion of a suspected scaphoid fracture by MRI will mean that the patient can avoid unnecessary immobilisation of the wrist with associated societal, financial and opportunity costs.
